# Evaluation of mycotoxin sequestering agents for aflatoxin and deoxynivalenol: an *in vitro* approach

**DOI:** 10.1186/2193-1801-3-346

**Published:** 2014-07-08

**Authors:** Changsu Kong, Seung Youp Shin, Beob Gyun Kim

**Affiliations:** Department of Animal Science and Technology, Konkuk University, Seoul, 143-701 Republic of Korea

**Keywords:** Mycotoxin, Swine, Mycotoxin adsorbent

## Abstract

An experiment was conducted to determine the efficacy of mycotoxin sequestering agents for binding or degrading aflatoxin B1 (AFB1) and deoxynivalenol (DON) by an *in vitro* method. Ten toxin binder products including 5 bentonite clays (bentonite A, B, C, D, and E), 2 cellulose products (cellulose A and B), a yeast cell wall, an activated charcoal, and a mixture product containing minerals, microorganisms, and phytogenic substances were used in this experiment. An *in vitro* procedure was used to mimic the digestive process in pigs. The binding ability for AFB1 of the cellulose products was less compared with the values of other sequestering products (*p* < 0.05). The percent adsorption of AFB1 by bentonite clays, cellulose products, yeast cell wall product, activated charcoal product, and the mixture product were 92.5 (average of 5 bentonite products), −13.5 (average of 2 cellulose products), 92.7, 100.2, and 96.6, respectively. The respective values for DON were 3.24, 11.6, 22.9, 14.4, and 4.3. In conclusion, most toxin sequestering agents used in the present study had potential to bind AFB1 rather than DON based on the *in vitro* study which simulated the pH condition of the gastrointestinal tract of pigs.

## Introduction

Dietary mycotoxins have been shown to cause detrimental effects in swine health and production. Aflatoxin and deoxynivalenol (DON) are produced by molds such as *Aspergillus* and *Fusarium*, respectively, and are frequently found in feedstuffs in swine diets (Council for Agricultural Science and Technology, 2003). Recent studies employing meta-analytical approach indicated that aflatoxin and DON depressed the growth performance of pigs (Andretta et al. 2012; Mok et al. 2013).

Several methods have been used to overcome detrimental effects of mycotoxins from contaminated feedstuffs. These include the thermal inactivation and irradiating as physical method, treatment with acid/base solutions, ozonation, and ammoniation as chemical method, and degradation of toxins by microorganisms as biological method (Diaz and Smith 2005). In feed industry, toxin sequestering agents have been frequently used because of its economic feasibility and suitability for nutritional perspective.

Several *in vitro* methods have provided a good idea of binding affinity and capacity, consequently have been used as a screening method for potential mycotoxin sequestering agents (Diaz et al. 2002; Marroquin-Cardona et al. 2009). However, these methods may not be directly applicable to pig diets because they did not use the successive incubation at different pH conditions similar to the intestinal environment of pigs. Thus, the objective of this experiment was to determine the binding efficacy of various sequestering agents to mycotoxins by an *in vitro* method which mimicked the gastrointestinal condition of pigs.

## Materials and methods

### Sequestering agents

Ten toxin binder products including 5 bentonite clays (bentonite A, B, C, D, and E), 2 cellulose products (cellulose A and B), a yeast cell wall product, an activated charcoal product, and a mixture product consisted of minerals, microorganism, and phytogenic substances were used in this experiment.

### Toxin preparation

The standard solution of AFB1 (2 μg/mL) and DON (100.2 μg/mL) in acetonitrile (Romer Labs Diagnostic GmbH, Tulln, Austria) were diluted to 10 and 250 ng/mL using distilled water, respectively. The quantification ranges of enzyme-linked immunosorbent assay (ELISA) kit used for analysis on AFB1 and DON were from 2 to 50 and 250 to 5,000 ng/mL, respectively.

### In vitro procedure

An *in vitro* procedure was modified from suggested *in vitro* digestion procedure which simulates the digestion procedure of pigs (Boisen and Fernández 1997). Each sample consisted of 2.5 mL of phosphate buffer (0.1 M, pH 6.0) and 0.5% suspension of each sequestering agent was transferred to 50 mL conical tube and 5 mL of diluted mycotoxin standard solution was added to the conical tube. For control treatment, 5 mL of phosphate buffer added. The pH was adjusted to pH 2.0 by adding 300 μL 1 M HCl for simulating pH in the stomach. Then each sample was incubated for 2 h in shaking incubator at 39°C. The incubation of samples was conducted in triplicates of each sequestering agent sample. After 2-h incubation, 1 mL of phosphate buffer (0.2 M, pH 6.8) was added to the conical tube. For simulating the conditions in the small intestine, 300 μL of 1 M NaOH was also added and incubated at pH 6.8 for 4 h. After incubation, the mixture was centrifuged and the supernatant was obtained for analysis of residual unbound AFB1 and DON. The AgraQuant® Aflatoxin B1 (COKAQ8000) or Deoxynivalenol (COKAQ4000) ELISA test kits (Romer Labs Inc., Singapore) were used to detect the residual unbound AFB1 or DON concentration, respectively.

### Calculations and statistical analyses

The percent adsorption of AFB1 and DON by sequestering agents was calculated using the following equation:


where IMT (ng/mL) is the initial amount of mycotoxin (AFB1 or DON) in the digestion conical tube; UMT (ng/mL) is the residual amount of unbound mycotoxin (AFB1 or DON) in the conical tube after digestion procedure.

Data were analyzed by MIXED procedure of SAS (SAS Inst. Inc., Cary, NC, USA). The model included the sequestering agent as a fixed variable. Differences among least squares means were determined by the PDIFF option with the Tukey’s adjustment. The significance was declared at an alpha-level of 0.05.

## Results and discussion

The percent adsorption of AFB1 by various sequestering agents is presented in Table 1. The residual amount of unbound AFB1 was greater in cellulose products treatment than in the rest of sequestering products (*p* < 0.05). The percent adsorption of AFB1 by bentonite clays, cellulose, yeast cell wall product, activated charcoal product, and the mixture product were 92.5 (average of 5 bentonite products), −13.5 (average of 2 cellulose products), 92.7, 100.2, and 96.6, respectively. The binding ability for AFB1 of the cellulose products was less compared with the values of other sequestering products (*p* < 0.05). With exception of the cellulose products, the percent adsorption of AFB1 by the sequestering agents used in the current study was generally high and this result agrees with the previous *in vitro* study in which binding ability of aforementioned products were over 90% (Diaz and Smith 2005). In addition, several *in vivo* study including pigs and broilers have shown that the sequestering agents ameliorated the detrimental effect of AFB1 (Schell et al. 1993; Miazzo et al. 2000; Raju and Devegowda 2000; Rosa et al. 2001). But, it is unclear why the percent adsorption of AFB1 by cellulose products was very low or negative. However, an overestimation of myxotoxins may occur if there are matrix effects of cellulose products on the detection of AFB1 because the target compounds are not antigens but mycotoxins in the present ELISA assay (Trucksess and Koeltzow 1995).

**Table 1:** ***In vitro*** percent adsorption of aflatoxin by various sequestering agents*

Sequestering agent	Amount of aflatoxin, ng/mL		Aflatoxin adsorption, %
	Initial	Unbound ^1^	
Control	10.00	10.00	0
Bentonite A		0.57^c^	94.3^a^
Bentonite B		1.91^c^	80.9^a^
Bentonite C		0.57^c^	94.4^a^
Bentonite D		0.08^c^	99.2^a^
Bentonite E		0.63^c^	93.7^a^
Cellulose A		13.09^a^	−31.0^c^
Cellulose B		9.60^b^	4.0^b^
Yeast cell wall		0.73^c^	92.7^a^
Activated charcoal		−0.02^c^	100.2^a^
Mixture^2^		0.34^c^	96.6^a^
SEM^3^	-	0.51	5.06
*p*-value	-	< 0.001	< 0.001

*Each least squares mean represents three observations.

^1^Calculated in comparison to the control treatment containing no sequestering agent.

^2^The mixture product consisted of minerals, microorganism, and phytogenic substances.

^3^Standard error of the means.

^a-b^Values within a column without a common superscript letter differ (*p* < 0.05).

The amount of residual unbound DON after digestion procedure was lower in yeast cell wall products (*p* < 0.05) than bentonite D but it did not differ from the values of other products (Table 2). The low DON-binding ability of sequestering agents in the present work agrees with the result from an *in vitro* assay study in which the potential binders including mineral clays, humic substances and yeast-derived products were tested for the binding ability of DON and zearalenone by using a conventional incubation followed by a specific bioassay for mycotoxin detection (Sabater-Vilar et al. 2007). The percent adsorption of DON by bentonite clays, cellulose, yeast cell wall product, activated charcoal product, and the mixture product were 3.24 (average of 5 bentonite products), 11.6 (average of 2 cellulose products), 22.9, 14.4, and 4.3, respectively. The sequestering ability for DON of yeast cell wall product was greater than that of bentonite D (*p* < 0.05). Most sequestering agents except cellulose products were relatively lower in ability for sequestering DON compared with AFB1 (average 7.2 vs. 94.0%). This result was in agreement with the result observed in the previous study (Avantaggiato et al. 2005). Bentonite has been known as a good and selective adsorbent for AFB1 rather than DON (Phillips 1999) and this has been shown in the reported *in vivo* study in which the addition of bentonite with 0.5% modified yeast cell wall to the naturally DON-contaminated diets did not play a significant role in detoxification of DON (Shehata et al. 2004). This was also in agreement with the binding activity of bentonite in the current study as well as the values from the previous *in vitro* study (Avantaggiato et al. 2004). In contrast, activated charcoal adsorbed 14.4% DON from 1.25 μg/mL toxin in the current study, whereas the respective value was 87.7% (average of 3 observations) from 2.00 μg/mL toxin in other *in vitro* study which used the dynamic *in vitro* model simulating the GI-tract conditions of pigs (Avantaggiato et al. 2004).

**Table 2:** ***In vitro*** percent adsorption of deoxynivalenol by various sequestering agents*

Sequestering agent	Amount of deoxynivalenol, ng/mL		Deoxynivalenol adsorption, %
	Initial	Unbound ^1^	
Control	250.00	250.00	0
Bentonite A		242.21^ab^	3.1^ab^
Bentonite B		243.57^ab^	2.6^ab^
Bentonite C		242.90^ab^	2.8^ab^
Bentonite D		247.40^a^	1.0^b^
Bentonite E		233.13^ab^	6.7^ab^
Cellulose A		234.28^ab^	6.3^ab^
Cellulose B		207.94^ab^	16.8^ab^
Yeast cell wall		192.85^b^	22.9^a^
Activated charcoal		213.94^ab^	14.4^ab^
Mixture^2^		239.32^ab^	4.3^ab^
SEM^3^	-	13.98	5.59
*p*-value	-	0.019	0.019

*Each least squares mean represents three observations.

^1^Calculated in comparison to the control treatment containing no sequestering agent.

^2^The mixture product consisted of minerals, microorganism, and phytogenic substances.

^3^Standard error of the means.

^a-b^Values within a column without a common superscript letter differ (*p* < 0.05).

In conclusion, the present study showed that most sequestering agents tested had sufficient potential to bind AFB1 rather than DON based on the *in vitro* experiment which mimicked the pH condition of the gastrointestinal tract of pigs.
